# Study of solid loading of feedstock using trimodal iron powders for extrusion based additive manufacturing

**DOI:** 10.1038/s41598-023-32095-5

**Published:** 2023-03-24

**Authors:** Heungseok Oh, Taehyeob Im, Jungsuk Pyo, Jai-sung Lee, Caroline Sunyong Lee

**Affiliations:** 1 Department of Materials and Chemical Engineering, Hanyang University, Ansan, 15588 Republic of Korea; 2 Solueta Co. Ltd. R&D Center, Hwaseong, 18544 Republic of Korea

**Keywords:** Materials science, Nanoscale materials

## Abstract

Volume loading of feedstock using trimodal iron (Fe) powders was investigated for the application of extrusion-based additive manufacturing (AM). Fe trimodal powder composed of nano, sub-nano, and micro particles was manufactured via the powder metallurgy process where small particles behave as rolling bearings among large particles, and thereby improving the flow characteristics of feedstock by minimizing friction among the particles. The flow behavior and microstructures of the monomodal feedstock were compared with those of the trimodal feedstock. We have confirmed that the critical powder loading of monomodal powder was measured to be 70 vol.% while trimodal powder showed up to 74 vol.%. Furthermore, trimodal feedstocks of 60, 65, 70, 75, and 80 vol.% Fe powder were prepared to determine the optimal powder content for sintering. As a result, the feedstock with powder content of 70 vol.% gave the highest sintered density of 92.32%, the highest Vickers hardness of 80.67 HV, with the smallest dimensional variation in shrinkage, proposing 70 vol.% of trimodal feedstock to be the suitable powder content for AM. Finally, its microstructural and mechanical comparison with 70 vol.% sintered part using monomodal Fe powder, showed that the sintered part using trimodal feedstock displayed higher hardness, uniform shrinkage as well as smaller grain size, confirming trimodal feedstock to be favorable for the application of extrusion-based AM.

## Introduction

Fused deposition modeling (FDM) is one of Additive Manufacturing (AM) technology that extrudes the material through the printing nozzle^1–5^. Unlike laser-based AM technologies [e.g., selective laser sintering (SLS), directed energy deposition (DED), and laser-engineered net shaping (LENS)], the FDM method uses inexpensive materials and equipment, and is very competitive in terms of energy consumption^6–8^. However, high-melting materials cannot be used since operating temperature of the commercially available FDM machine is about 300 °C^9,10^. One way to print high-melting metallic materials using FDM technology is to use a metal powder–binder mixture (feedstock) which has been used in conventional metal injection molding^11,12^. The feedstock could have highly viscous behavior due to the binder having low softening temperature. For FDM, the feedstock is heated until the material is softened and extruded through a nozzle of the printer fabricating a green part of the desired shape. The feedstock composed of powder–binder mixture enables a part to be produced at a temperature much lower than the melting point of the metal^13–15^.

To be used in automotive, aerospace, military, and architectural applications, high sintering density, high mechanical properties, and uniform shrinkage during sintering are required. Recent studies have shown that as the solid loading increases, these characteristics have improved^16,17^. However, as the solid loading increases, the friction between the powders rises, leading to an increase in viscosity, which makes extrusion through the nozzle very difficult. To achieve smooth extrusion, it is essential to study the critical solid loading which is the allowable highest powder content while minimizing its increase in viscosity. Since metal feedstock has been used in injection molding for many years, extensive research has been carried out on the critical solid loading as Table 1 summarizes previous studies on the critical loading of feedstock used in injection molding.

**Table 1:** Literature search on critical solid loading for metal injection molding.

No	Material	Powder characteristic	Binder system	Critical loading	Published year	References
1	17-4PH	D_50_ = 6.9 μm	PP, PW, SA	60 vol.%	2020	^18^
2	316L	D_50_ = 5.96 μm (water atomized)	PMMA, PEG, SA	61.5 vol.%	2009	^19^
3	316L	D_50_ = 8 μm (irregular)	PP, CW, PE, SA	64 vol.%	2009	^20^
4	316L	D_50_ = 10.21 μm	LDPE, HDPE, PW, SA	62 vol.%	2019	^21^
5	Fe powder	Fe nano powder D_50_ = 100 nm Fe micro powder D_50_ = 3 μm	PP, PE, PW, SA	58 vol.%	2019	^22^
6	Fe–2Ni–2Cu gas atomized powder	D_50_ = 4.03 μm	PP, PE, PW, SA	60 vol.%	2021	^23^
7	Fe-50Ni	Fe and Ni mixed powder D_50_ = 4.98 μm	PP, PE, PS, PW, SA	62 vol.%	2014	^24^
8	Fe-50Ni	Atomized Fe powder D_50_ = 3.0 μm, Atomized Ni powder D_50_ = 2.1 μm	PP, PW, SA	60.49 vol.%	2020	^25^
9	Ti-6Al-4V powder	D_50_ = 13.42 μm	LDPE, PW	65 vol.%	2013	^26^
10	Ti-6Al-4V powder	Ti powder D_50_ = 26.5 μm, 60Al-40 alloy powder D_50_ = 20.6 μm	PP, PE, PW, SA	64 vol.%	2017	^27^
11	Ti-6Al-4V powder	D_50_ = 51.8 μm	PEG, PVB, SA	68 vol.%	2019	^16^

Based on these studies done by Askari et. al and Singh et al. etc., it was found that the flowability of the material is reduced as the cavitation is induced in the feedstock itself when using powders greater than 65 vol.%, making extrusion very difficult and causing defects in the molded part. However, Studies done by Thavanayagam et al. achieved critical solid loading to be 68 vol.% using powder with 51.8 $\mathrm{\mu m}$ in size^16^. However, it is known that large powder size is not appropriate for extrusion based additive manufacturing since that can eventually clog the nozzle^28^.

The feedstock used in extrusion-based additive manufacturing needs to have lower solid loading to be viscous enough for printing. Suwanpreecha et al. summarized various papers on the production of parts using metal extrusion-based additive manufacturing. The materials commonly used in metal extrusion-based additive manufacturing include stainless 316L, 17-4PH, Ti-6AL-4V, and Cu. The solid loading used in several studies generally falls within the range of 55–60 vol.%, while cases using powder loading over 60 vol.% are not common due to the high viscosity making it difficult to extrude through a nozzle^29^. Therefore, the powder loading used in metal extrusion-based additive manufacturing is about 5 vol.% lower than the ones used for the injection molding.

**Figure 1:**
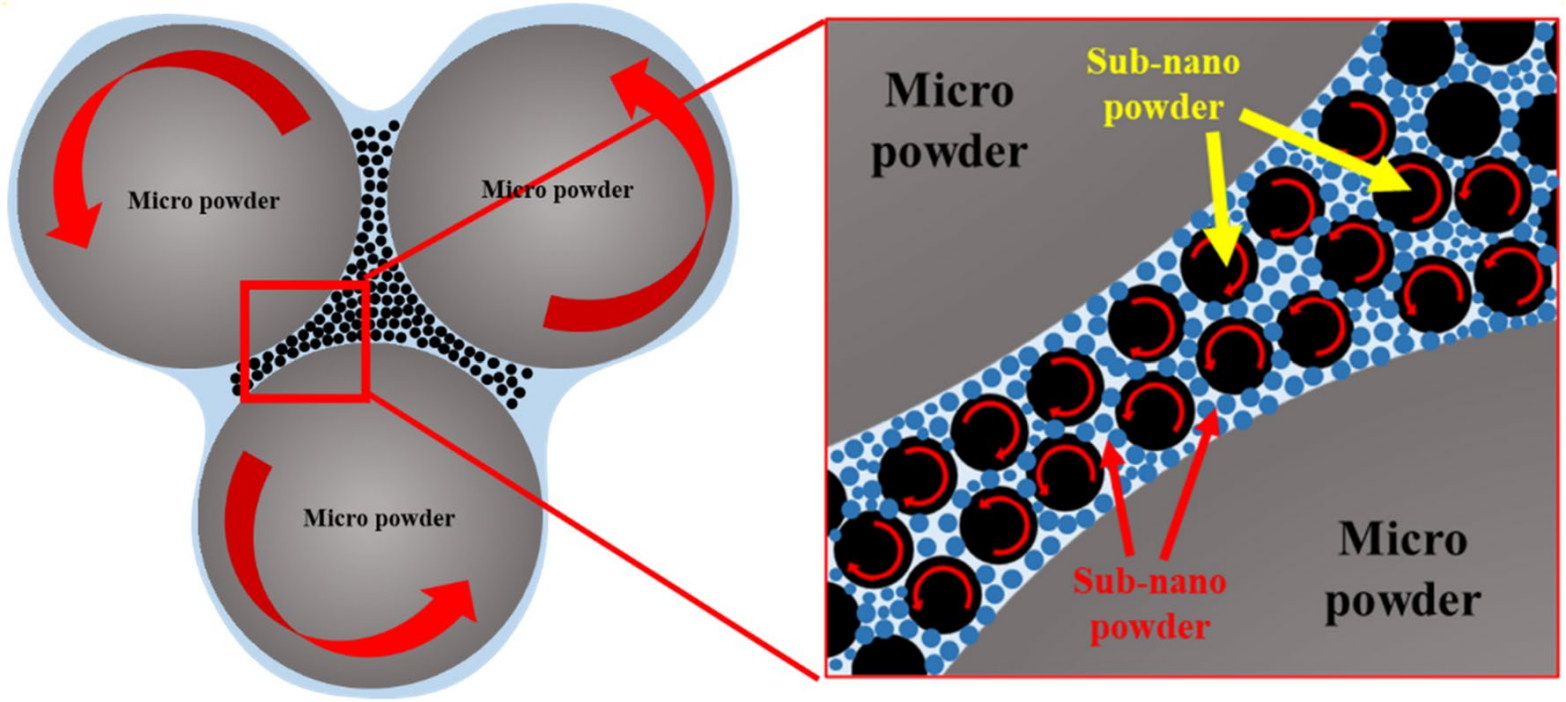
Schematic diagram showing rolling bearing effect among micro-sized, sub nano-sized, and nano-sized powder.

In this study, trimodal powder was introduced to improve its critical powder loading for extrusion-based additive manufacturing to overcome this problem stated above. Trimodal powder is composed of nano, sub-nano, and micro particles where nano/sub nano particles in the trimodal feedstock are packed between single-sized micro particles, improving packing efficiency and acting as rolling bearings to minimize friction forces between particles. In this way, The lubrication effect of nano-sized particles can enhance the critical solid loading of the feedstock, resulting in improved sintering density as well as uniform sintering shrinkage^30^. The schematic of rolling bearing effect is shown in Fig. 1. Moreover, nano/sub nano sized particles can form nano grains with dense grain boundaries after sintering. Therefore, mechanical properties can be improved through the pinning effect, inhibiting migration of the micro grain boundaries^22^.

Therefore, we have fabricated a feedstock composed of trimodal Fe powder for the application of Extrusion-based AM to investigate its effect of particle size distribution (PSD) on sintering shrinkage and mixing torque in comparison to those using monomodal sized Fe feedstock. Moreover, the different solid loading ranging from 60 to 80 vol.% trimodal feedstock was studied to determine the suitable powder content of Fe trimodal feedstock to achieve the highest sinter density for the application of Extrusion-based AM.

## Experimental procedure

**Figure 2:**
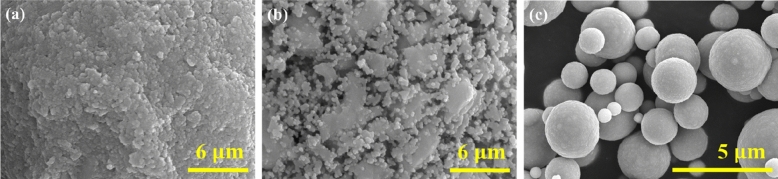
Scanning electron microscopy images of (**a**) iron oxide nanopowder, (**b**) hydrogen-reduced iron nanopowder agglomerates showing bimodal particle size distributions, and (**c**) spherical Fe powder.

Alpha-iron oxide (α-Fe_2_O_3_) micropowder (99.9% pure; Kojundo Chemical, Japan) was pulverized using a high-energy bead mill (Nanointech, Korea) to obtain homogeneously milled iron oxide nanopowder, as follows. The micropowder (500 g) was milled at 2400 rpm for 20 h using methanol and zirconia beads having a diameter of 1 mm as milling media. The milling process converted the micro-sized α-Fe_2_O_3_ powder into a nanosized iron oxide slurry, which was subsequently dried using a spray dryer (Mihyun Engineering, Korea). Scanning electron microscopy (SEM) image of the resulting nanopowder is presented in Fig. 2a. The dried iron oxide powder was reduced to pure iron (Fe) powder via heat treatment in a hydrogen atmosphere (99.999%), as the temperature was increased from room temperature to 550 °C at a rate of 10 °C/min over 1 h. During this process, Ostwald ripening occurred due to size difference of the Fe nanoparticles^31^ whereby smaller particles decreased in size and merged with larger ones. The heat treatment resulted in an Fe powder with a bimodal PSD. Images of the Fe nanoparticles after the heat treatment are presented in Fig. 2b; bimodal PSDs are clearly evident.

The bimodal Fe nanopowder (25 vol.%) was mixed with spherical Fe powder (75 vol.%) (99.9% purity; 5–10 μm; Avention, Korea) in a conventional tubular mixer (HANtech, Korea) to form a trimodal powder. SEM image of spherical Fe powder is presented in Fig. 2c. Since Fe nanopowder is readily oxidized in air due to its high surface area, the powder mixture was coated with stearic acid (95.0% purity; Samchun, Korea). The trimodal Fe powder was then demonstrated into a trimodal feedstock by mixing with a wax-based binder system composed of paraffin wax (75 vol.%) and stearic acid (25 vol.%) as follows; Mixing was done using a twin-screw mixer (CW Brabender Instruments, USA) operating at 75 °C and 60 rpm. Mixing torque as a function of powder content was used to assess the flow behavior of the monomodal and trimodal feedstocks. In each case, the initial powder content was 60 vol.% and the mixing torque was measured as 2 vol.% of powder was added incrementally.

Rheological property of the trimodal feedstock was determined according to the mixing torque obtained during its mixing process. The shear rate was calculated by Eq. (1),

1 $$\dot{\gamma }=\frac{{2}^{2(1+1/n)}\pi N}{n} \frac{{\beta }^{2/n}}{{\left(1+\beta \right)}^{2/n}({\beta }^{2/n}-1)},$$

where $\dot{\gamma }$ is the shear rate, n is the flow behavior index, *N* is the rotor speed of the mixer, and $\beta$ is the ${R}_{e}/{R}_{i}$ ratio, and Eq. (2):

2 $$\eta \left(\dot{\gamma }\right)=\frac{2\Gamma }{\pi L{\left({R}_{e}+{R}_{i}\right)}^{2}\left(1+{G}^{n+1}\right)\dot{\gamma }},$$

where $\eta$ is the shear viscosity, $\Gamma$ is the mixing torque, *L* is the length of the rotor, ${R}_{i}$ is the equivalent internal radius, ${R}_{e}$ is the equivalent external radius, and *G* is the gear ratio^32^. Mixing torque was measured while changing the mixing speed from 4 to 10, 30, and 60 rpm at 75 °C. The dependence of the shear viscosity on shear rate was then established using Eqs. (1) and (2) ^32,33^.

Specimens with the desired shape were printed using homemade screw-type extrusion-based AM equipment. Feedstock was mechanically grounded as powder to supply through the hopper of AM machine for homogeneous extrusion. Hollow shaped specimens were printed using the G-code of Cura software (Ultimaker, USA). Layer height was determined based on nozzle diameter of 1 mm. The printing temperature was 75 °C, which was higher than the melting point of the binder; additional printing conditions such as printing speed, flow, and initial layer thickness are experimentally optimized which are provided in Table 2.

**Table 2:** Printing condition.

Printing condition	Value
Nozzle size (mm)	1
Layer height (mm)	0.9
Fill density (%)	0
Printing speed (mm/s)	30
Nozzle temperature (°C)	75
Flow (%)	20
Initial layer thickness (mm)	0.6

Here, flow at 20% means fraction of the 100% extruded amount to prevent excessive extrusion which can cause non-uniform printing. Fill density is defined as 100% when solid cylinder is printed. In this study, we had hollow structure so that its fill density of 0% was entered. Finally, nozzle diameter of 1 mm was used during printing.

**Figure 3:**
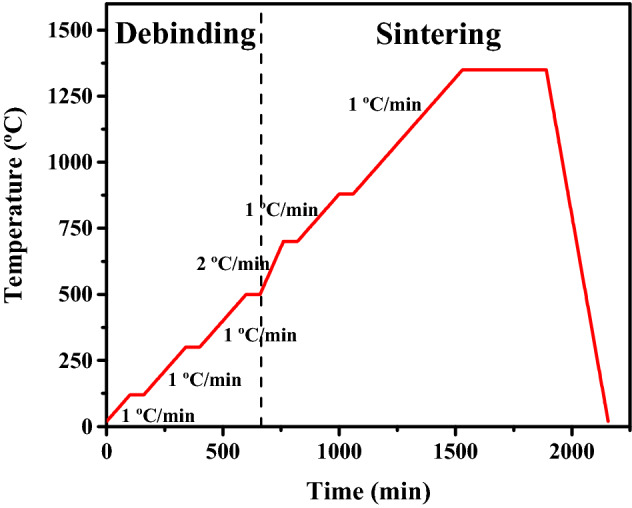
Debinding and sintering profile.

The debinding process was performed for removal of binders prior to sintering process. Debinding used ramped temperature profiles from room temperature to 120 °C over 1 h (1 °C/min), 300 °C over 1 h (1 °C/min), and 500 °C over 1 h (1 °C/min). Sintering process was carried right after the debinding process through series of steps by heating upto 700 °C over 1 h (2 °C/min), 880 °C over 1 h (1 °C/min), and 1350 °C over 6 h (1 °C/min), in a tube furnace (Myeongseong Engineering, Korea) under a mixed-gas atmosphere of H_2_/Ar at 5/95 v/v to prevent oxidation (Fig. 3). We have used a mixed–gas atmosphere of H_2_/Ar instead of 100% H_2_ for the reasons of safety, economic efficiency, and ease of mass production to broaden readers.

The Vickers hardness (FV-700, Future-Tech, Japan) measurement as well as microstructural observation using SEM was done for the final sintered part while the porosity was measured by image J analysis. The shrinkage in external diameter, thickness and height for the final sintered part, was calculated by measuring its dimensional change before and after the sintering was done. Sintered density was measured by the Archimedes method (Mettler Toledo, USA). The measurement was performed with ethanol as fluid at room temperature. Finally, these properties were compared with those for the sintered part using monomodal sized particles that are 5–10 μm in diameter (Fig. 1c).

## Results and discussion

**Figure 4:**
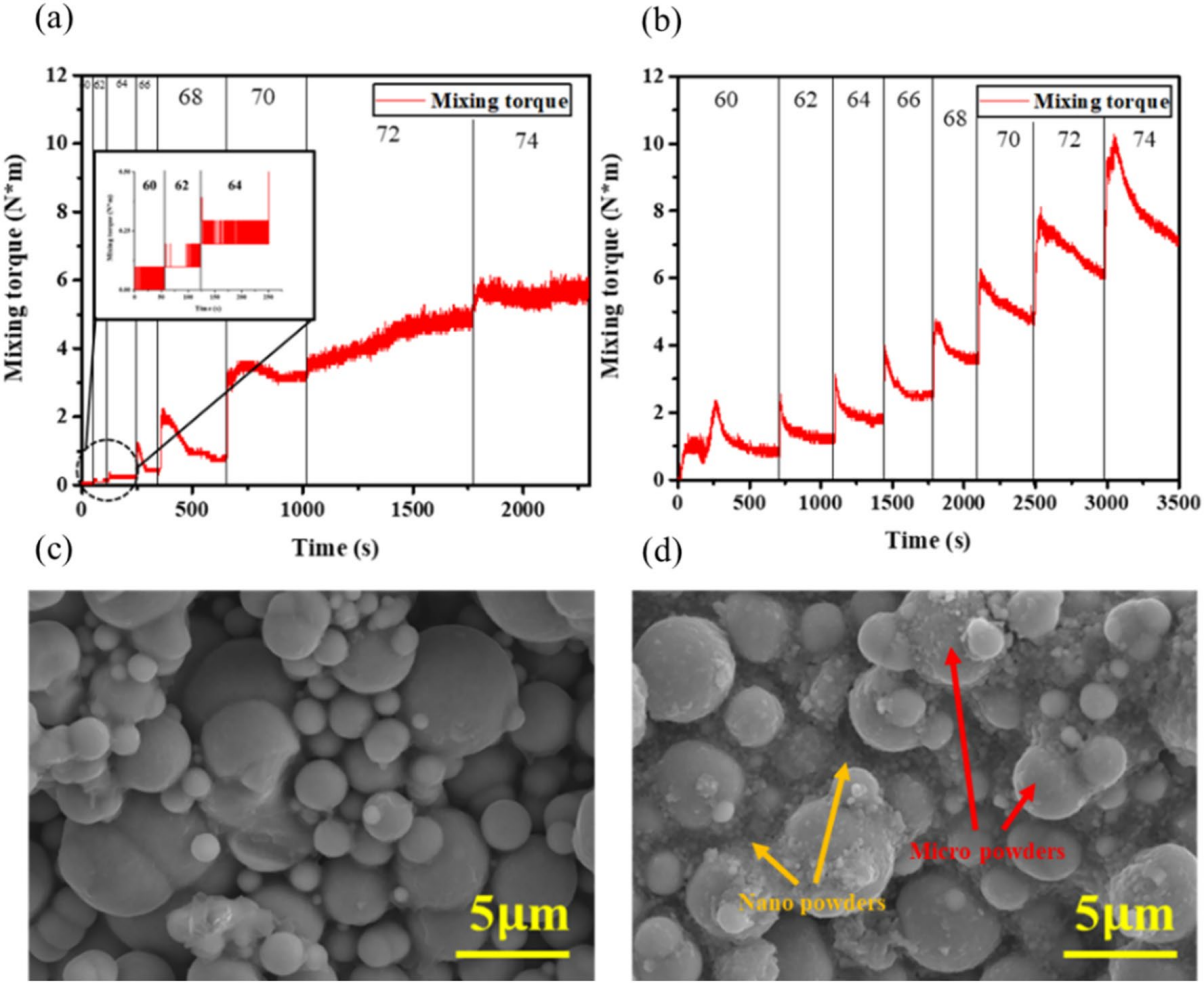
Mixing torques measurement results ranging from 60 to 74 vol.% of (**a**) monomodal and (**b**) trimodal feedstocks. SEM images of 74 vol.% of (**c**) monomodal and (**d**) trimodal feedstocks.

Figure 4a presents a mixing torque graph of the monomodal feedstock whose mixing torque stabilizes in a relatively short time. The monomodal feedstock consisted only of larger particles, which packed to form relatively large voids that facilitated binder penetration. The stabilizing time steadily increased with adding powder, describes gradually reaching to critical solid loading. When the solid loading reaches up to 70 vol.%, the mixing torque did not stabilize due to insufficient binder which can reduce the frictional force between Fe particles^34^.

In Fig. 4b, the trimodal feedstock took a relatively long time for the mixing torque to stabilize. It was significantly more difficult for the binder to penetrate the much smaller voids between the nanoparticles. However, critical loading was improved from the nanoparticles by increasing the packing density. Therefore, the mixing torque of feedstock was stabilized up to 74 vol.% feedstock.

**Figure 5:**
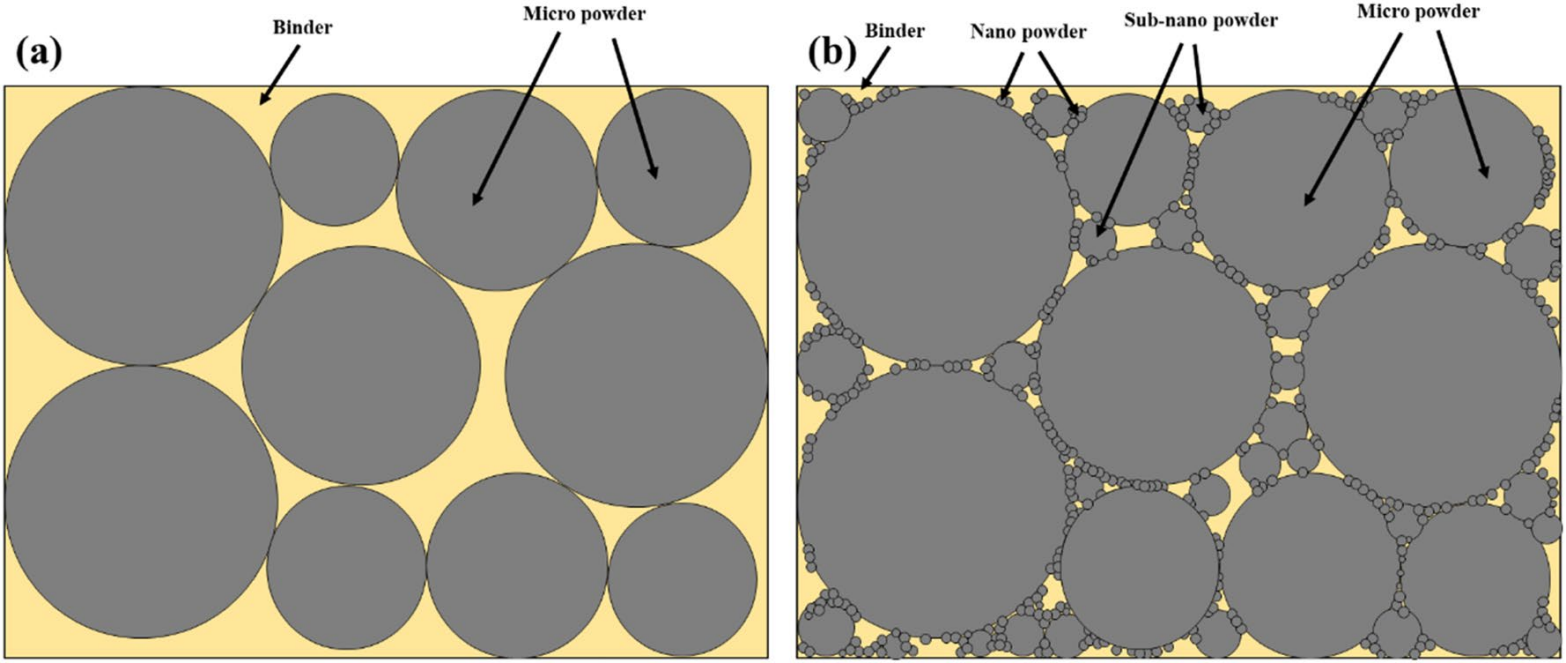
Schematic comparing its microstructure of (**a**) monomodal feedstock and (**b**) trimodal feedstock.

Figure 4c,d presents SEM images of the monomodal and trimodal 74 vol.% feedstocks. Relatively large amounts of voids were evident among the monomodal microparticles due to the lack of binder (Fig. 4c). On the other hand, no void was observed in the trimodal feedstock (Fig. 4d). Figure 5 shows schematic diagram illustrating different microstructures of monomodal and trimodal feedstocks. In Fig. 5a with monomodal feedstock, the micro particles are closely packed inside the feedstock, but due to those inevitable voids formed among monomodal sized powders, relatively large amount of binder is required. In contrast, there are nano powder and sub-nano powder filling the spacing among the micro particles for trimodal feedstock shown in Fig. 5b. Therefore, the amount of binder loading could be decreased compared to those for monomodal feedstock, resulting in improvement of critical loading of powder, in agreement with mixing torques measurement results shown in Fig. 4.

In conclusion, the trimodal feedstock required relatively less binder than the monomodal feedstock. In addition, small particles reduce friction among the Fe particles, which enabled a higher critical solid loading of the feedstock. Thus, a feedstock with high powder content can be manufactured using a trimodal Fe powder, and debinding and sintering will provide parts with good mechanical properties.

**Figure 6:**
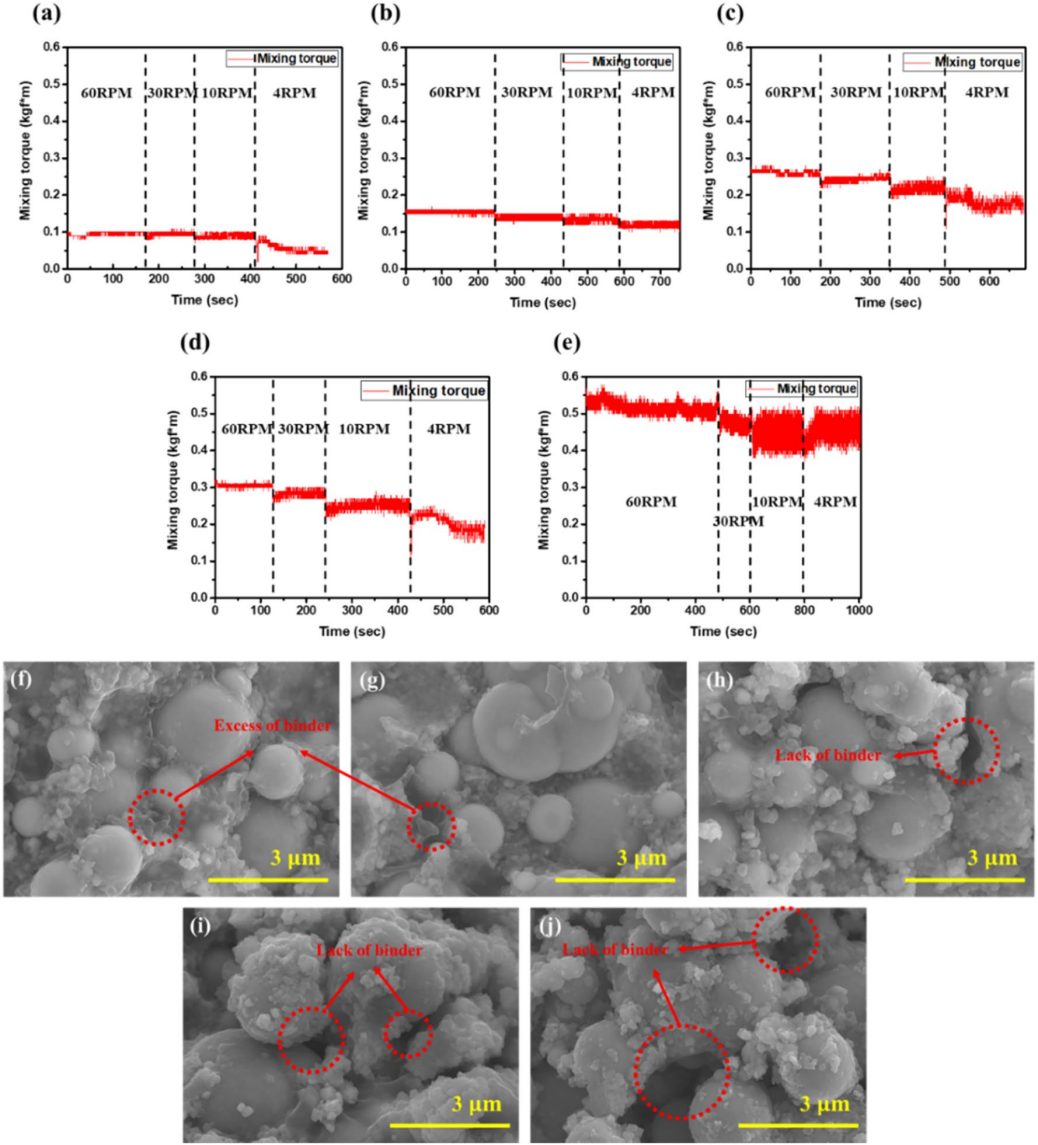
Mixing torque measurement results of varying trimodal feedstocks at different mixing speeds for (**a**) 60 vol.% (**b**) 65 vol.% (**c**) 70 vol.% (**d**) 75 vol.%, and (**e**) 80 vol%. SEM images of corresponding trimodal feedstocks for (**f**) 60 vol.%, (**g**) 65 vol.%, (**h**) 70 vol.%, (**i**) 75 vol.%, and (**j**) 80 vol.%.

The optimal solid loading is the highest powder content within a range having flow characteristics suitable for extruding defect-free structures. To determine the optimal powder content of the trimodal feedstock, the mixing torque was measured as a function of mixing speed for feedstocks with different powder contents. Figure 6a–e show that the mixing torque increased with increasing powder content. This is a predictable result attributable to the increasing frictional force between powder particles. The variation in mixing torque of the 60 vol.% feedstock was very small (Fig. 6a). Small variation indicates that the feedstock is stable and has homogeneously incorporated the binder. However, the variation in mixing torque gradually increased with increasing powder content. At 80 vol.%, the variation was substantial due to trapped air and insufficient binder. Free Fe powder prevented the formation of a homogeneous powder–binder mixture. Such binder-poor mixtures can result in nonuniform extrusion during the manufacturing process^25,33,35^. Feedstocks exhibit pseudoplastic fluid behavior, i.e., shear viscosity increases with decreasing shear rate. The mixing torque of feedstock should increase according to the mixing speed. However, the mixing torque of the 80 vol.% feedstock did not increase as the mixing speed as reduced from 10 to 4 rpm; this was due to free Fe powder in the inhomogeneous mixture^34,36^.

The SEM images of Fig. 6f–j show the microstructure of the feedstock prepared with different powder contents. The nanoparticles were located between microparticles, while binder was mixed between them. The image of the 60 vol.% feedstock in Fig. 6f shows a large amount of binder, which decreased density. However, the amount of residual binder decreased with increasing powder content. Images of the 75 and 80 vol.% feedstocks (Fig. 6i,j) show individual particles due to insufficient binder^36,37^. In contrast, the 70 vol.% feedstock had relatively few defects, such as insufficient or excess binder, and images indicated that the powder and binder were uniformly blended.

**Figure 7:**
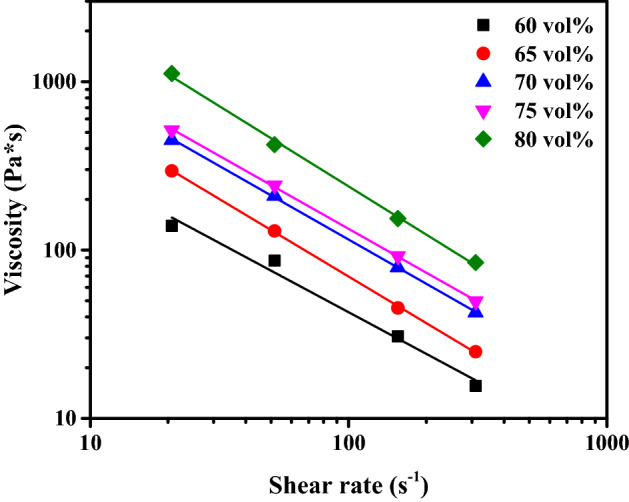
Relationship between the shear rate and viscosity of the trimodal feedstock with varying solid loadings.

The viscosity of the feedstock is an important variable when extruding a material; the lower the viscosity, the easier it is to print. The mixing torque of each solid loading of feedstock and other torque rheometer variables measured while changing the mixing speed were substituted into Eqs. (1) and (2) to quantify the effect of shear rate on feedstock viscosity; the results are summarized in Fig. 7. At a given shear rate, the viscosity of the feedstock increased with increasing powder content. At a given powder content, the viscosity decreased with increasing shear rate.

**Figure 8:**
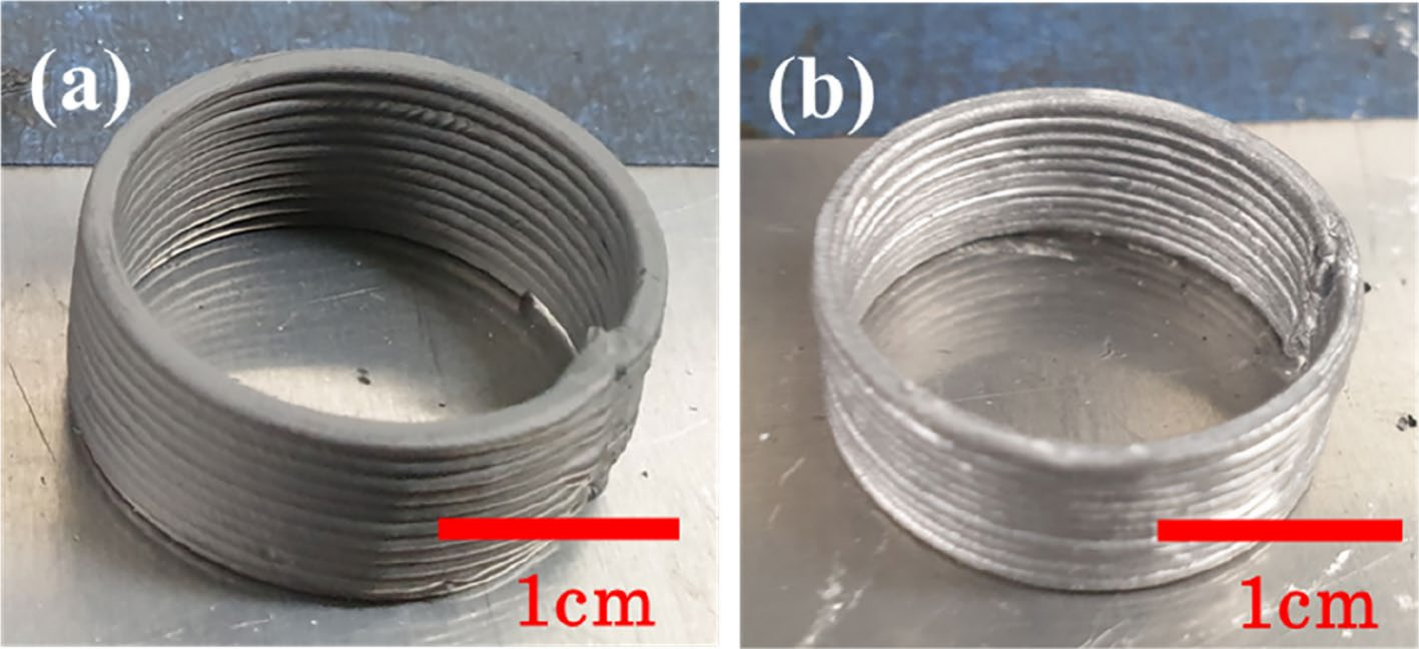
Photographs of the 70 vol.% part (**a**) additively manufactured and (**b**) sintered.

When printing the feedstock, the viscosity of the feedstock for each solid loading is obtained by calculating the shear rate at the nozzle. The shear rate of the nozzle is established from Eq. (3) which shows the relationship between shear rate and flow rate inside of the nozzle.

3 $$\dot{\gamma }=\frac{4Q}{\pi {R}^{3}}.$$

where Q is the volumetric feed rate, and R is the wall radius. For the nozzle size of 1 mm in diameter and printing speed of 30 mm/s, the shear rate was calculated to be 240 s^−1^. The viscosities were calculated to be 20.76, 31.05, 53.63, 62.50, and 103.51 Pa s for 60, 65, 70, 75 and 80 vol.% trimodal feedstocks respectively. These feedstocks were processed into the parts using an Extrusion based AM machine. As a result, the feedstocks ranging from 60 to 75 vol.% were extruded from the nozzle to form the shape presented in Fig. 8a. In contrast, uniform extrusion was difficult when 80 vol.% feedstock was extruded due to high shear viscosity of 103.51 Pa s so that it was impossible to manufacture the specimen with the desired shape. Therefore, we can assume that the maximum printable viscosity range to be somewhere between 62.50 and 103.51 Pa s.

**Figure 9:**
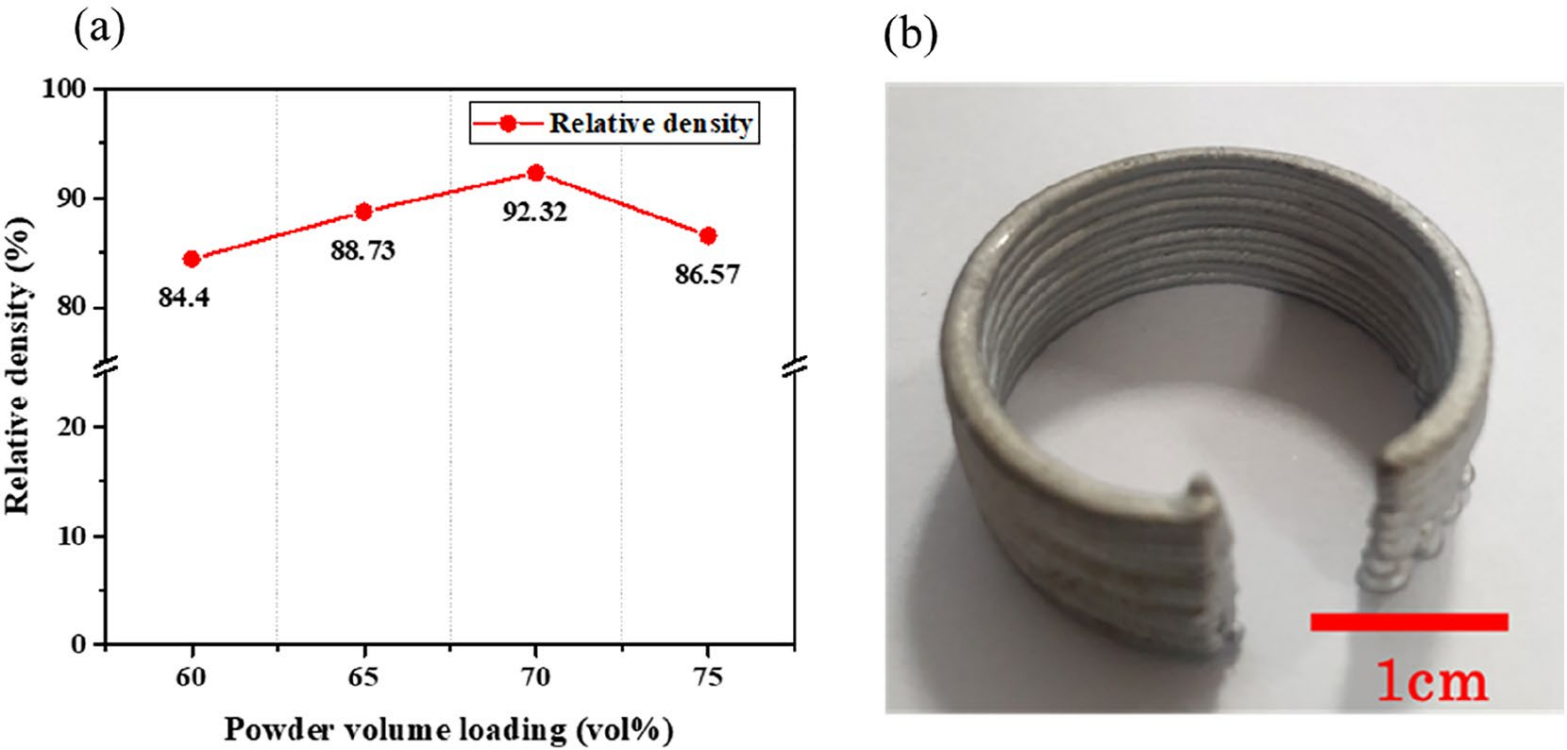
(**a**) Relative density of sintered specimens as a function of powder loading and (**b**) photograph of the sintered 75 vol.% part.

Debinding and sintering were performed under optimized heat-treatment conditions. Photograph of the sintered part is shown in Fig. 8b. Figure 9a shows the relative density of the sintered specimens for the different solid loadings. The excess binder in the 60 vol.% feedstock became pores during the debinding process; these pores could not be fully removed during the sintering process since they were trapped within the sintered body. The relative density was the lowest for the 60 vol.% feedstock and increased with increasing powder content up to 70 vol.%, where the maximum relative density of 92.32% was achieved. Increasing the loading further to 75 vol.% resulted in a reduction in density, likely due to formation of pores caused by excessive powders relative to the amount of binders, as those observed in Fig. 6i. Furthermore, the 75 vol.% specimens fractured during the sintering process (Fig. 9b). The shrinkage that occurred during sintering, which led to the development of stresses in the parts, and the formation of pores, which acted as defects, eventually caused the parts to fracture.

**Figure 10:**
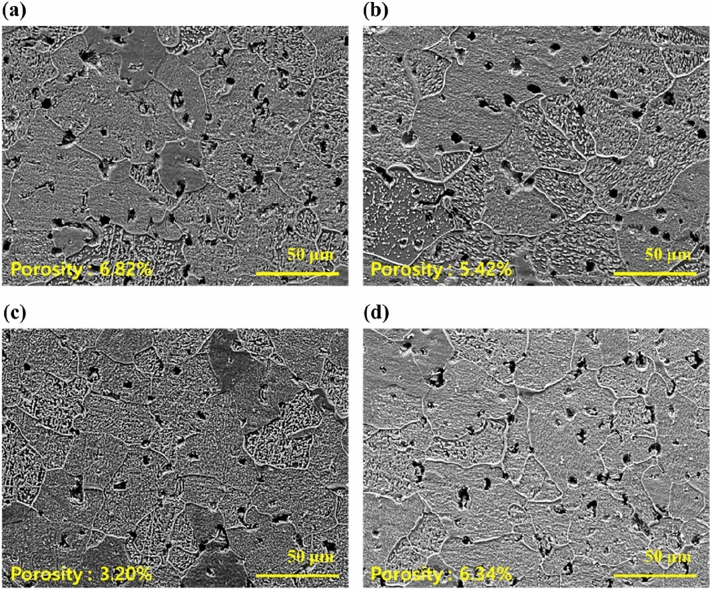
SEM images of sintered Fe trimodal feedstocks with (**a**) 60 vol.%, (**b**) 65 vol.%, (**c**) 70 vol.% and (**d**) 75 vol.%.

Furthermore, the cross-sectional SEM images of the sintered samples with varying powder content are shown in Fig. 10. As shown in the figure, microstructure of the 60 vol.% sample (Fig. 10a) shows the highest porosity of 6.82%, attributed to the debinding process of the excess binder shown in Fig. 6f. The excess binder in the 60 vol.% feedstock appears to transform into pores during debinding and they did not seem to be entirely eliminated during sintering. However, porosity gradually decreases with increasing powder content (Fig. 10b,c), reaching its lowest value of 3.20% at 70 vol.% (Fig. 10c). In contrary, further increasing the powder content to 75 vol.% (Fig. 10d) resulted in higher porosity due to lack of binder shown in Fig. 6i.

Table 3 compares the grain size and Vickers hardness of the sintered trimodal feedstocks with different powder content. As a result, average grain size shows uniform distribution of about 35 μm, irrespective of the powder content. For hardness test, 70 vol.% trimodal feedstock showed the highest hardness value of 80.67 HV. Therefore, 70 vol.% trimodal feedstock appears to be the optimized solid loading based on the microstructural observations and hardness measurement results,.

**Table 3:** Grain size and Vickers hardness measurement results of the sintered trimodal feedstock with 60 vol.%, 65 vol.%, 70 vol.% and 75 vol.% (grain size was determined using ImageJ analysis and hardness measurement was done on 10 different spots for each sample).

	Trimodal 60 vol.%	Trimodal 65 vol.%	Trimodal 70 vol.%	Trimodal 75 vol.%
Grain size (μm)	35.85 $\pm$ 2.22	37.74 $\pm$ 4.33	33.43 $\pm$ 1.60	34.22 $\pm$ 0.67
Hardness (HV)	69.50 $\pm$ 4.71	78.12 $\pm$ 7.11	80.67 $\pm$ 6.45	65.67 $\pm$ 0.49

**Figure 11:**
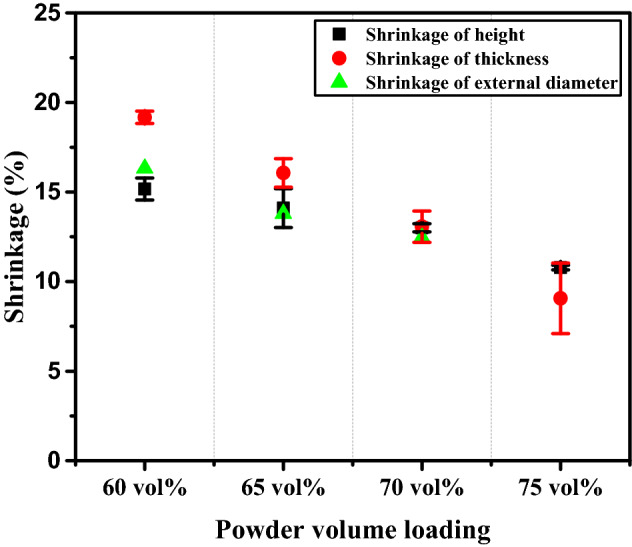
Dimensional shrinkage of sintered specimens as a function of powder loading. The measurement of the shrinkage in height, thickness and external diameter was done five times for each sample.

Nonuniform distribution of binder in a feedstock can lead to local shrinkage that exceeds the average shrinkage values for the specimen. Figure 11 presents the shrinkages in height, thickness, and outer diameter, expressed as the percentage change in dimension from the green to the sintered part. In any dimension, the shrinkage decreased with increasing powder content. Sintered specimens prepared from the 60 vol.% feedstock displayed nonuniform shrinkage across the dimensions (height, thickness and external diameter) due to excessive binder, as observed in the SEM image of Fig. 6f.

Increasing the feedstock powder content to 65 vol.% resulted in less differences in shrinkage across each dimension, and at 70 vol.% the shrinkages were still lower, and displayed less variation because of the uniform mixing of metal powder and binder in the feedstock. The specimen fractured when the 75 vol.% feedstock was used, which prevented calculation of the change in outer diameter (Fig. 9b). However, less shrinkage in the height and thickness dimensions was observed. In summary, sintered 70 vol.% trimodal feedstock was found to be the optimized sample showing the highest sintered density, least porosity, highly uniform shrinkage and the highest hardness value among samples with different solid loading.

**Figure 12:**
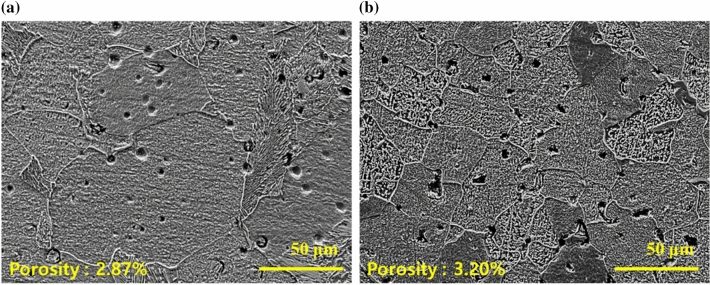
SEM images of sintered 70 vol.% parts using (**a**) monomodal and (**b**) trimodal sized Fe powder.

Using 70 vol.% trimodal feedstock, we have compared mechanical property and microstructures with the sintered parts using monomodal Fe powder. The cross-sectional SEM images of microstructures for both sintered samples (Fig. 12) show that the porosities for both samples seem to be similar. Observing SEM image of the sintered trimodal sized Fe feedstock, smaller grain size was observed throughout the sample than those for the sintered monomodal Fe powder (Fig. 12a,b). We had observed that the trimodal sized sample exhibited smaller grain size of 33.43 ± 1.60 μm while the monomodal sized sample with its grain size of 44.30 ± 3.76 μm in average was obtained (Table 4). Moreover, sintered trimodal Fe feedstock with smaller grain size resulted in higher hardness value due to grain boundary strengthening effect caused by nano particles inhibiting coarsening of the micro powder^22,38^.

**Table 4:** Measurement results of average grain size and Vickers hardness of the 70 vol.% sintered part using monomodal vs. trimodal powder (grain size was determined using ImageJ analysis and hardness measurement was done on 10 different spots for each sample).

	Monomodal	Trimodal 70
Grain size (μm)	44.30 $\pm$ 3.76	33.43 $\pm$ 1.60
Hardness (HV)	57.03 $\pm$ 2.18	80.67 $\pm$ 6.45

**Figure 13:**
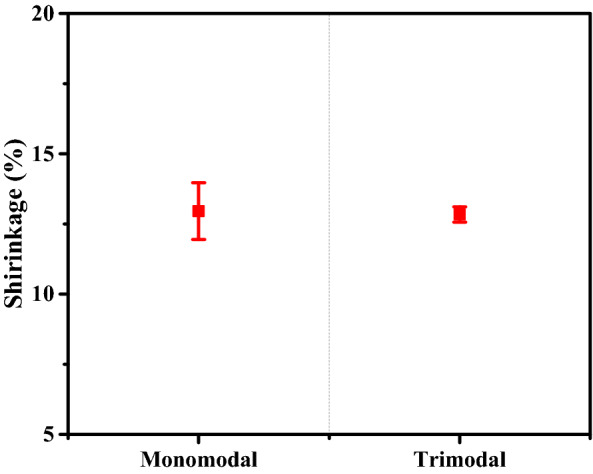
Comparison of dimensional shrinkage of 70 vol. % sintered specimens using trimodal vs. monomodal powder (average value was taken for shrinkage of height, thickness, and outer diameter for each sample).

Finally, the dimensional shrinkage of 70 vol.% Sintered specimens using monomodal vs. trimodal sized Fe powder was calculated by measuring changes in height, thickness, and outer diameter of each sample to plot their average values for comparison (Fig. 13). As a result, both samples showed its shrinkage value of approximately 13%. However, the monomodal sample showed larger deviation than that of the trimodal sample. Therefore, it was confirmed that the nano-particles in the trimodal feedstock play a crucial role as a skeleton between the micro-particles, preventing anisotropic shrinkage. This ultimately helps in preventing defects and cracks in the final sintered part, leading to a higher level of dimensional accuracy.

## Conclusion

In this study, a binder–powder feedstock was selected as a material for extrusion-based AM. Using trimodal Fe particles improved solid loading. Rheological testing and microstructural observations confirmed that the trimodal feedstock enabled higher critical solid loading of 74 vol.% compared with that of the monomodal feedstock with 70 vol.%.

To determine the optimal powder content for sintering, the mixing torque, viscosity, microstructure, sintered density, and shrinkage were determined for trimodal feedstocks having powder contents ranging from 60 to 80 vol.%. The 80 vol.% feedstock was difficult to extrude through the printing nozzle due its high shear viscosity. The 75 vol.% feedstock showed relatively stable mixing torque but some large microvoids were present among particles. As a result, the specimen fractured during sintering due to the presence of these voids, resulting in relatively low sintered density. In contrast, 60 and 65 vol.% feedstock displayed low mixing torque with the presence of excess binder. This feedstock was easy to extrude due to its low mixing torque, but yielded sintered parts of low density and anisotropic shrinkage. As the 70 vol.% trimodal feedstock resulted in higher mixing torque with shear viscosity compared with those for 60 and 65 vol.% feedstocks, the relative density after the sintering was resulted in 92.32%, the highest among the various feedstocks tested. Moreover, the shrinkage was isotropic across the height, thickness, and outer diameter dimensions. In conclusion, 70 vol.% was determined to be the suitable critical loading for trimodal feedstock with the smallest dimensional variation in shrinkage during sintering for the application of extrusion-based AM. Moreover, its microstructural and mechanical comparison with 70vol% sintered part using monomodal Fe powder, showed that the sintered part using trimodal feedstock displayed higher hardness, uniform shrinkage as well as smaller grain size, confirming trimodal feedstock to be favorable for the application of extrusion-based AM.

## Supplementary Information

Supplementary Information.

## Data Availability

The datasets used and/or analysed during the current study available from the corresponding author on reasonable request.
