# Supplementary material for: Study of solid loading of feedstock using trimodal iron powders for extrusion based additive manufacturing
Source: Sci Rep. 2023 Mar 24;13:4819. doi: 10.1038/s41598-023-32095-5 (PMC10038991; doi:10.1038/s41598-023-32095-5)
Supplement: Supplementary file 1 — Supplementary Information. [file 41598_2023_32095_MOESM1_ESM.pdf]

Study of Solid loading of Feedstock using trimodal iron powders for Extrusion based Additive Manufacturing

Heungseok Oh a, Taehyeob Im a, Jungsuk Pyo a,b, Jai-sung Lee a, Caroline Sunyong Lee a,*

a Department of Materials and Chemical Engineering, Hanyang University, Ansan 15588, Republic of Korea

b Solueta Co. Ltd. R&D Center, Hwaseong 18544, Republic of Korea

*Corresponding author

Caroline Sunyong Lee, sunyonglee@hanyang.ac.kr

Supplementary Information

Table S1. Sintering conditions.

Sample	Sintering temperature (°C)	Heating rate (°C/min)	Time (h)
#1	1,300	10	3
#2	1,350	10	3
#3	1,400	10	3
#4-1	1,250	5	3
#4-2	1,300	5	3
#4-3	1,350	5	3
#5	1,300	10	6
#6	1,250	5	6

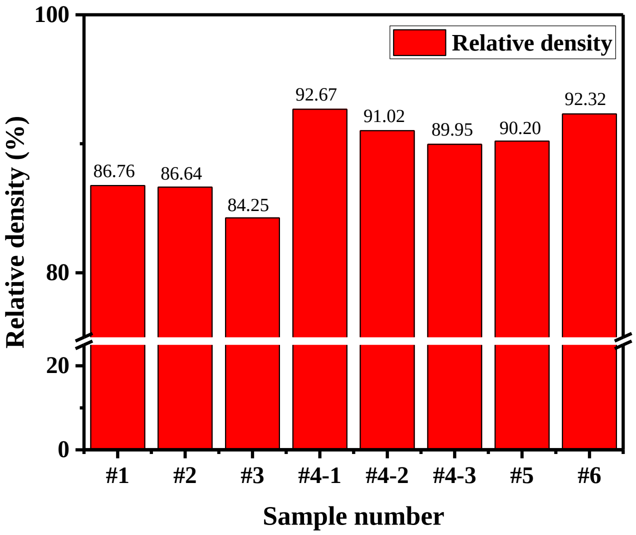

Figure S1. Relative density of parts sintered according to heating profile.

Sintering was carried out under the conditions listed in Table S1 to optimize the parameters for sintering. Specimens typically densify with increasing sintering temperature. However, a different trend was observed with our samples (Figure S1). The first (#1, #2, and #3) and second (#4-1, #4-2, and #4-3) groups of samples were sintered for the same times but at different temperature ramping rates, i.e., 10°C/min and 5°C/min, respectively. Higher sintering temperature led to the densification of nanoparticles near the surface in the initial stages of sintering, and pores remained within the parts. Sample #1 from the first group and sample #4-1 from the second group showed the highest density at the lowest sintering temperature within each group. Thus, the feedstocks containing nanoparticles responded differently to the sintering temperature.

Comparing pairs #1, #4-2, and #2, #4-3 revealed the effect of heating rate on sintered density. A low

heating rate significantly improved the relative density. Comparing #1 with #4-2, the improvement was 4.26%, while comparing #2 with #4-3, it was about 3.31%. The heating rate had a significant effect on relative density because the nanoparticles in the feedstocks had a higher sintering driving force, which resulted in closed pores due to sintering of the specimen surface. Comparing #1 with #5 revealed the effect of sintering time on density. Sample #5 was sintered for 3 h longer than composition #1, which resulted in ~3.44% higher density. This finding is consistent with pore closure during extended sintering, which accordingly improved density [1].

The density of a sintered specimen is related to its porosity. The optical microscope images in Figure S2 and porosities in Table S2 revealed the pore structure obtained under each sintering condition. Figure S2 (a), (b), and (c) shows the pore structures of samples #1, #2, and #3, respectively; changes in pore structure with sintering temperature are clearly evident. Table S2 indicates that composition #1 had the lowest porosity within the first group of samples. This was attributed to nanoparticles in the trimodal feedstock, which facilitated the trapping of pores within the sample during the sintering process. As the sintering temperature increased, so too did the size of the pores, resulting in an irregular structure. Such nonuniform pore structure may deteriorate the mechanical properties [2].

Figure S2 (d), (e), and (f) also show differences in pore structure according to sintering temperature. As shown in Figure S2 (a), (b), and (c), the porosity was lowest when the temperature was low, and increased with temperature. Figure S2 (a), (e) confirmed the change in pore structure with heating rate. Although specimen #4-2, sintered under a low heating rate, showed lower porosity at 13.53%, there was no significant difference in pore size. Figure S2 (a), (g) confirmed the change in pore structure with sintering time. When the sintering time was 3 h, the porosity was 16.32%, while after 6 h, the porosity was 15.12%. Although sintering time had some effect on internal pore reduction, it was less influential than sintering temperature or heating rate. Figure S2 (h) shows the pore structure of a specimen sintered under condition #6; it displayed the lowest porosity. The porosity data in Table S2 correspond well with the relative density data. The sintering condition of 1,250°C, 5° C/min, and 6 h was optimal, providing the lowest porosity and improved relative density.

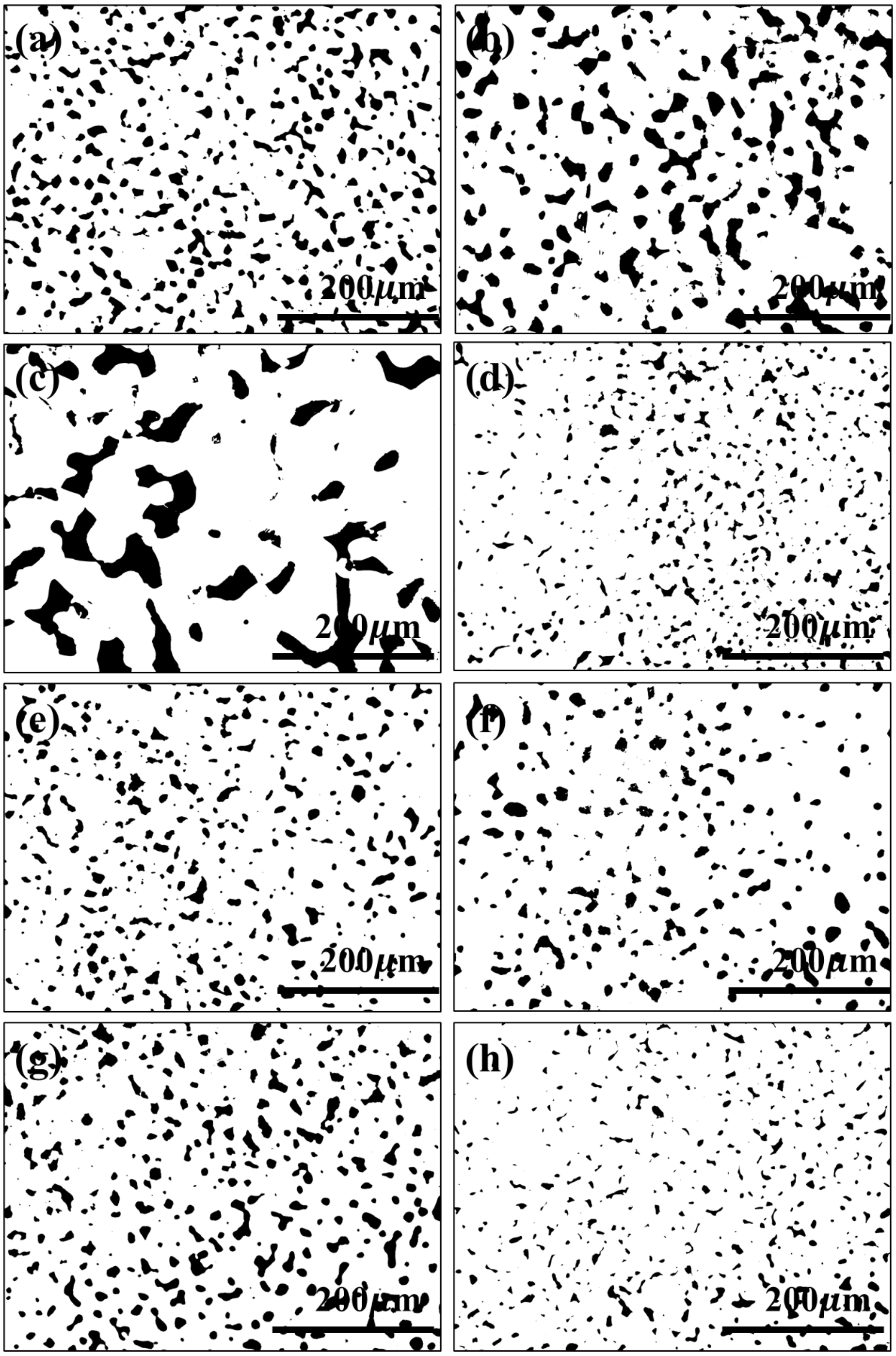

Figure S2. Optical microscopy images of sintered specimens showing the pore structure. (a) #1, (b) #2, (c) #3, (d) #4-1, (e) #4-2, (f) #4-3, (g) #5, and (h) #6.

Table S2. Porosity of parts sintered under different conditions.

	#1	#2	#3	#4-1	#4-2	#4-3	#5	#6
Porosity	16.32	18.62	18.57	8.48	9.70	9.80	15.12	6.72

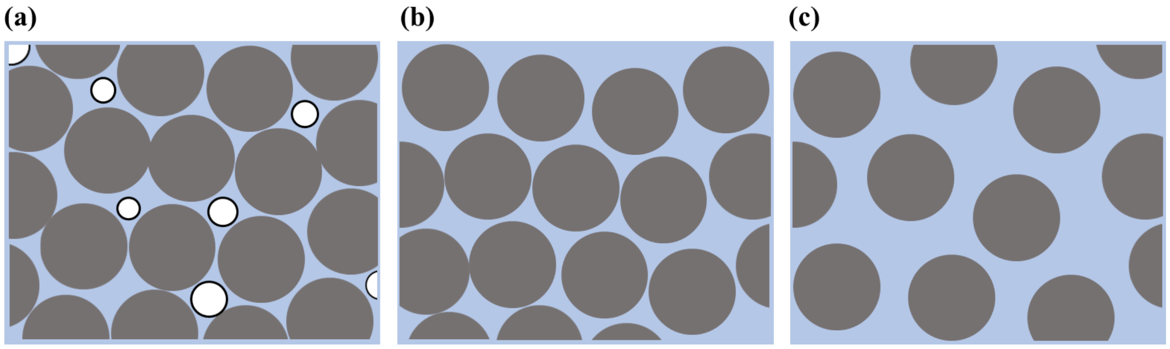

Figure S3. Schematic illustrations of particle packing in feedstocks. (a) Excess of binder, (b) at critical volume loading, (c) excess binder [3].

Three cases with different powder loadings are shown in Figure S3. Particles are not closely packed when there is excess binder (Figure S3 (c)). Combining the powder with insufficient binder results in lower density and larger dimensional shrinkage. The particles in the feedstock can be densely packed with the optimal balance of powder and binder. This microstructure maximizes the green density, which greatly affects the density of the sintered specimen. However, the addition of metal powder to the critical loading condition generates voids between the particles (Figure S3 (a)). Consequently, feedstocks with insufficient binder have inhomogeneous compositions and display poorer rheological properties, and the sintered specimens contain pores. Therefore, determining the critical loading of a feedstock is essential to fabricate parts with good properties.

Reference

[1] J.-P. Choi, J.-S. Park, E.-J. Hong, W.-S. Lee and J.-S. Lee "Analysis of the rheological behavior of Fe trimodal micro-nano powder feedstock in micro powder injection molding". vol. 319, pp. 253-260, 2017, doi: 10.1016/j.powtec.2017.06.056

[2] C. Ji, N. Loh, K. Khor and S. Tor "Sintering study of 316L stainless steel metal injection molding parts using Taguchi method: final density". vol. 311, pp. 74-82, 2001, doi: 10.1016/S0921-5093(01)00942-X

[3] Y. Li, L. Li and K. Khalil "Effect of powder loading on metal injection molding stainless steels". vol. 183, pp. 432-439, 2007, doi: 10.1016/j.jmatprotec.2006.10.039

The English in this document has been checked by at least two professional editors, both native speakers of English. For a certificate, please see:

http://www.textcheck.com/certificate/eRC4oo
